# 2009 Pandemic Influenza A (H1N1) Virus Outbreak and Response – Rwanda, October, 2009–May, 2010

**DOI:** 10.1371/journal.pone.0031572

**Published:** 2012-06-26

**Authors:** Justin Wane, Thierry Nyatanyi, Richard Nkunda, Joseph Rukelibuga, Zara Ahmed, Caitlin Biedron, Adeline Kabeja, Marie Aimée Muhimpundu, Alice Kabanda, Simon Antara, Olivier Briet, Jean Baptiste Koama, André Rusanganwa, Odette Mukabayire, Corine Karema, Pratima Raghunathan, David Lowrance

**Affiliations:** 1 King Faisal Hospital, Kigali, Rwanda; 2 Center of Treatment and Research on HIV/AIDS, Malaria, Tuberculosis and Other Epidemic Diseases, Ministry of Health, Kigali, Rwanda; 3 National Reference Laboratory, Kigali, Rwanda; 4 Influenza Division, Centers for Disease Control and Prevention (CDC), Kigali, Rwanda; 5 Division of Global HIV/AIDS, Centers for Disease Control and Prevention (CDC), Kigali, Rwanda; 6 African Field Epidemiology Network, Kigali, Rwanda; 7 World Health Organization, Kigali, Rwanda; National Institutes of Health, United States of America

## Abstract

**Background:**

In October 2009, the first case of pandemic influenza A(H1N1)pdm09 (pH1N1) was confirmed in Kigali, Rwanda and countrywide dissemination occurred within several weeks. We describe clinical and epidemiological characteristics of this epidemic.

**Methods:**

From October 2009 through May 2010, we undertook epidemiologic investigations and response to pH1N1. Respiratory specimens were collected from all patients meeting the WHO case definition for pH1N1, which were tested using CDC’s real time RT-PCR protocol at the Rwandan National Reference Laboratory (NRL). Following documented viral transmission in the community, testing focused on clinically severe and high-risk group suspect cases.

**Results:**

From October 9, 2009 through May 31, 2010, NRL tested 2,045 specimens. In total, 26% (n = 532) of specimens tested influenza positive; of these 96% (n = 510) were influenza A and 4% (n = 22) were influenza B. Of cases testing influenza A positive, 96.8% (n = 494), 3% (n = 15), and 0.2% (n = 1) were A(H1N1)pdm09, Seasonal A(H3) and Seasonal A(non-subtyped), respectively. Among laboratory-confirmed cases, 263 (53.2%) were children <15 years and 275 (52%) were female. In total, 58 (12%) cases were hospitalized with mean duration of hospitalization of 5 days (Range: 2–15 days). All cases recovered and there were no deaths. Overall, 339 (68%) confirmed cases received oseltamivir in any setting. Among all positive cases, 26.9% (143/532) were among groups known to be at high risk of influenza-associated complications, including age <5 years 23% (122/532), asthma 0.8% (4/532), cardiac disease 1.5% (8/532), pregnancy 0.6% (3/532), diabetes mellitus 0.4% (2/532), and chronic malnutrition 0.8% (4/532).

**Conclusions:**

Rwanda experienced a PH1N1 outbreak which was epidemiologically similar to PH1N1 outbreaks in the region. Unlike seasonal influenza, children <15 years were the most affected by pH1N1. Lessons learned from the outbreak response included the need to strengthen integrated disease surveillance, develop laboratory contingency plans, and evaluate the influenza sentinel surveillance system.

## Introduction

Rwanda is a landlocked, low-income country, situated in East Africa with an estimated 2010 population of 10.4 million. With 350 people per km^2^, it is the most densely populated country in Africa. It is estimated that 57.5% of the population is below 20 years of age. Females account for 52.3% of the population with an average life expectancy of 53.3 years compared to 49.4 years for males [1]. Rwanda has an equatorial climate with moderate temperatures and two rainy seasons, from March through June and from October through December [2].

Prior to the onset of the 2009 Pandemic Influenza A(H1N1)pdm09 (pH1N1) in North America, the Rwandan Ministry of Health (MOH) in collaboration with the U.S. Centers for Disease Control and Prevention established an influenza sentinel surveillance system (ISS) in July 2008 to understand the epidemiology of seasonal influenza and monitor for the emergence of a novel influenza strain with pandemic potential. Since the occurrence of the outbreak of pH1N1 in Mexico in April 2009, the MOH developed a pandemic operational plan in order to minimize the impacts of the pandemic [3]–[5]. After initial cases of A(H1N1)pdm09 were reported in Kenya, Tanzania and Uganda in June and July, 2009 respectively [6], [7], the MOH and their partners conducted a tabletop exercise in August 2009 to test the preparedness and readiness of the country to respond to the pandemic.

In Rwanda, the first case was identified in early October 2009 as a 42 year old female Rwandan who traveled back to Kigali after a short visit to the US. She presented without symptoms at the airport and developed an influenza-like illness (ILI) on October 4, 2009. Two of her children attending primary school fell ill on October 6 and 7, 2009. Healthcare providers at.

Hospital A, a tertiary referral facility in Kigali where the index case and her children had sought treatment became ill October 8, 2009. Samples collected on October 8, 2009 from the index case tested positive for pH1N1 virus by real time reverse transcriptase (rRT-PCR) at the National Reference Laboratory (NRL). The result was communicated by the MOH to the public on October 9, 2009.

Following this first laboratory confirmed case, the MOH established an inter-agency.

Multidisciplinary Emergency Working Group (EWG) to lead the outbreak investigation and response. We describe the clinical and epidemiological characteristics of this outbreak and response in Rwanda from October 2009 through May 2010.

## Methods

### Influenza Sentinel Surveillance Sites

**Figure 1:**
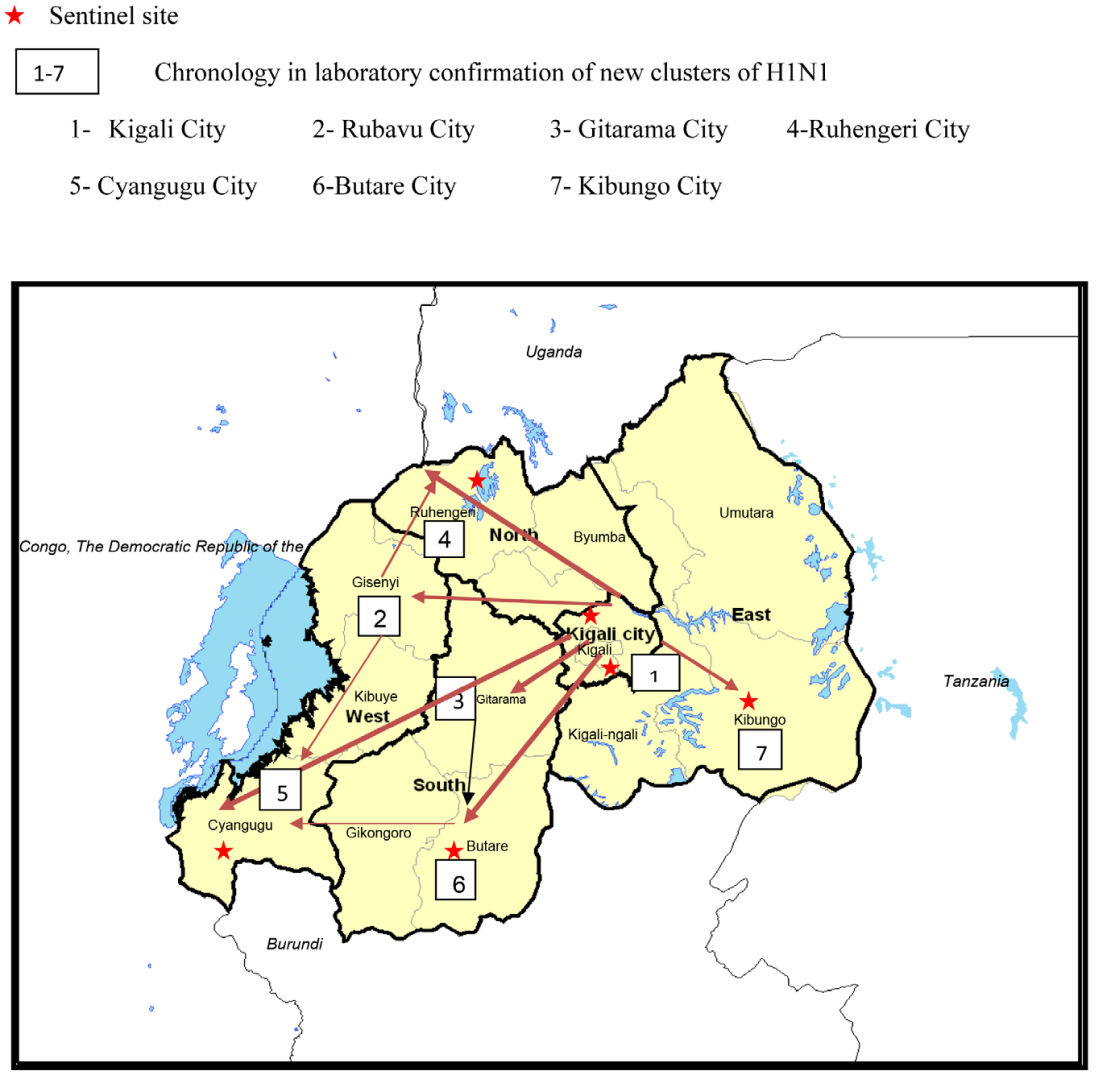
Location of Influenza Surveillance Sentinel sites and documented pH1N1 virus spread in Rwanda. From October 2009 - May 2010.

Since July 2008, an influenza sentinel surveillance system (ISS) had been established in six public hospitals (Figure 1), including two referral and four district hospitals. One referral hospital and one district hospital were located in the capital city of Kigali while three district hospitals and one referral hospital were located in the country’s other four provinces. The catchment area of each district hospital was approximately three hundred thousand persons, while the catchment area of the referral hospitals was the entire country. The selection was contingent upon the site’s capacity to collect and ship samples to the National Reference Laboratory (NRL) in Kigali and the site’s interest to participate in the ISS program. Each hospital had pediatric and adult inpatient wards as well as ambulatory care services, all of which participated in the surveillance program.

The ISS systematically identified Influenza-Like-Illness (ILI) and Severe Acute Respiratory Illness (SARI) cases at the selected sentinel sites from July 2008 to September 2009, according to the World Health Organization (WHO) case definitions. An ILI case was defined as an outpatient with fever (≥38°C) and cough or sore throat in absence of specific diagnosis with the onset of symptoms less than three days. A SARI case in “adults” was defined as a hospitalized patient 5 years and above with fever (≥38°C), cough, and shortness of breath or difficulty breathing with the onset of symptoms less than seven days. A SARI case in children was defined as a hospitalized patient 2 months to less than 5 years old, with cough or difficulty breathing, and at least one respiratory danger sign (unable to drink or breastfeed, lethargic, vomits everything, convulsions, nasal flaring, grunting, oxygen saturation <90%, chest indrawing, stridor in a calm child, tachypnea) with onset of symptoms less than seven days.

All eligible SARI cases in inpatient wards and the first two ILI cases per day from outpatients ward were included in the surveillance. Children aged less than 2 months were excluded.

For each enrolled SARI and ILI case, a questionnaire was completed that included demographic, clinical, and epidemiological information. In addition, a nasopharyngeal (NP) and oropharyngeal (OP) sample were collected by a trained nurse/laboratory technician. Swabs were placed in cryovials with 1 ml of viral transport media (VTM) and stored at 4°C for a maximum of 72 hours until they could be shipped to the NRL. At the NRL, RNA extractions were performed on specimens prior to freezing at −70°C using the QI Aamp Viral RNA Isolation Kit (QIAGEN, Valencia, CA, USA). Extracted RNA was amplified using Access RT-PCR System (Promega, Madison, WI, USA). Specimens were tested by real-time reverse transcriptase polymerase chain reaction (rRT-PCR) for presence of influenza A and B viruses using standard rRT-PCR procedures [3]–[5].

### Surveillance during the Containment Phase of the pH1N1 Outbreak Response

After the index case of pH1N1 was identified, the Emergency Working Group (EWG) revised the World Health Organization (WHO) case definitions for SARI and ILI in use at sentinel sites to include risk factors for pH1N1. The EWG also updated existing case investigation forms for enhanced ILI and SARI surveillance at sentinel and non-sentinel hospitals, and developed new data collection tools for outbreak investigation including a line list, pH1N1 contact monitoring form, and laboratory testing register.

On October 10^th^, 2009, the EWG began contact tracing to monitor for ILI and development of SARI symptoms in households of laboratory confirmed cases, schools attended by the index case’s children, the offices where the index case worked, Hospital A, where the index case had been treated, as well as among airplane passengers who traveled on the same flight as the index case. A contact was defined as a person who had close contact (within one meter) with a laboratory-confirmed case at any time during illness. A laboratory-confirmed case of pH1N1 virus infection was defined as a person with an acute febrile respiratory illness with laboratory confirmed non-seasonal influenza A(H1N1)pdm09 virus infection by real-time reverse transcriptase–polymerase chain reaction (rRT-PCR) at the NRL.

As part of contact tracing, a suspected case of pH1N1 was defined as any contact meeting WHO ILI or SARI case definitions with onset of symptoms within 7 days of close contact with a laboratory confirmed case of pH1N1 virus infection. All suspect pH1N1 cases had nasopharyngeal (NP) and/or oropharyngeal (OP) swabs taken by the laboratory technician of the outbreak investigation team. Specimens were then tested for pH1N1 and other influenza types and subtypes at the National Reference Laboratory using rRT-PCR assay based on the 2009 CDC guidelines for detection of A(H1N1)pdm09 virus using ABI 7500 Standard (Applied Biosystems Incorporation) [8].

Specimens were processed in batches of 24 specimens with a turnaround time of 12–24 hours. External quality assurance was provided by the Kenya Medical Research Institute-CDC laboratory in Nairobi, Kenya. Gradually, as the laboratory diagnosed new confirmed pH1N1 cases, new contact lists were established by the EWG in Kigali City. This practice was discontinued when the transmission generation was unknown and community transmission appeared widespread. On October 27, 2009, a foreign national residing in Gisenyi city in the Western Province of Rwanda and who recently arrived to Rwanda from the US developed an ILI. Samples collected from this case tested positive for pH1N1 virus infection on October 30^th^, 2009. This was the first laboratory-confirmed case outside of Kigali that was not epidemiologically linked to known cases or their contacts.

On November 6, 2011, pH1N1 positivity was confirmed among two foreign nationals attending solidarity camps in Muhanga District, South province. They arrived in Rwanda on November 1^t^, 2011 from Europe, also considered the external setting of likely transmission.

Laboratory-confirmed cases with no links to known cases or their contacts, or with history of external travel, were increasingly documented across the country, indicating widespread community transmission of pH1N1. Thus, on November 16, 2009, the EWG decided to transition from the initial containment phase to a mitigation phase. The containment phase lasted until November 16, 2009 and was characterized by intensive contact tracing, laboratory testing of suspect cases, mass communication, oseltavimir (Tamiflu®) distribution to suspect and laboratory-confirmed cases, and school closure [9]–.

### Surveillance during the Mitigation Phase of the pH1N1 Outbreak Response

Mitigation phase activities included decentralization of outbreak management to all 30 district hospitals, targeted laboratory testing of suspect pH1N1 cases, restrictive use of oseltamivir for most at risk laboratory-confirmed cases only, and enhanced surveillance.

**Figure 2:**
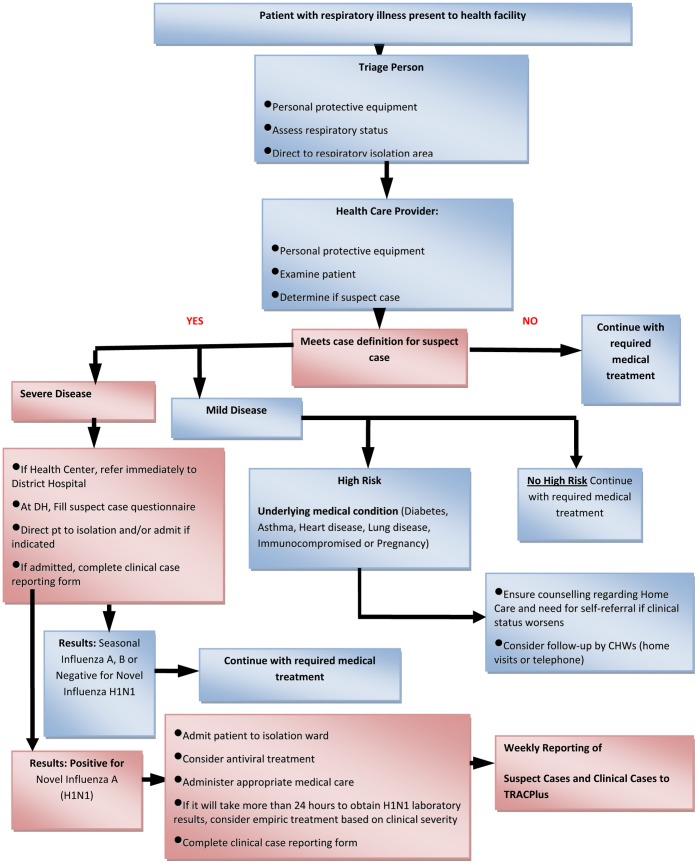
Clinical and laboratory testing algorithm used during the mitigation phase. From November 16, 2009– May 31, 2010.

In the context of community transmission of pH1N1 in the country, an increase in new cases of suspect pH1N1 was expected. With the limited laboratory capacity, NRL would not be able to test every suspect case. In view of these limitations, the EWG advised on the following and proposed an A(H1N1)pdm09 Laboratory testing algorithm (Figure 2);

1. Not all cases in a “new suspect cluster” of pH1N1 were tested. In a district where pH1N1 transmission had not previously been confirmed, about 1–5% of the suspected cases in a cluster had specimens collected from the severely sick (with a maximum of 10 samples from the biggest cluster) and all the others were line-listed as possible cases.

2. If a suspect case was severely ill or in a high-risk group, i.e. pregnant women, chronic respiratory problems (e.g. asthma, chronic bronchitis, cardiac problems, HIV/AIDS, cancer patients, diabetic, <5 yrs old) a specimen was taken.

3. Not all cases of pH1N1 required oseltamivir; treatment was given only to the severe cases and those at high risk of complications e.g. pregnant women, children under 5 years or those with an underlying medical condition like diabetes, asthma, heart disease, lung disease, or immuno-compromising illness. No prophylaxis with oseltamivir was recommended in any circumstances.

4. Due to community transmission of influenza A/H1N1 the EWG recommended community management and home based care of suspect mild cases of pH1N1. Thus, only severe cases were admitted, and. all other cases were managed conservatively as for seasonal influenza.

5. There was inadequate rationale and capacity for mass screening of institutions/schools for pH1N1, but in institutions where suspect cases occurred for the first time, a few specimens (not to exceed ten) were taken for testing.

### Data Analysis

Epi-Info version 3.5.1 (Centers for Disease Control and Prevention, Atlanta, Georgia) and Intercooled Stata® (StataCorp LP, College Station, Texas) were used to analyse the surveillance data. Outbreak investigation data were analyzed in Excel. Demographic, clinical and epidemiological characteristics of influenza cases between inter-pandemic and pandemic periods and the characteristics of positive and negative cases and virus circulation during the pandemic period in Rwanda from July 2008 to May 2010 were analyzed.

### Ethics Statement

The outbreak investigation and response conformed to the Helsinki Declaration and to local legislation. The activity was deemed non-research by the Rwanda National Ethics Committee and the U.S. Centers for Disease Control and Prevention.

## Results

### Sentinel Surveillance of Influenza Prior to the Pandemic

From July 2008 to September 2009, a total of 659 respiratory specimens were tested, of which 297 (45.1%) were ILI and 362 (54.9%) were SARI cases. Among SARI cases, 30.7% (111/362) were SARI Adult and 69.3% (251/362) were SARI Child cases. The ratio of females to males was 1.4 for ILI cases and 0.98 for SARI cases. The median age was 21.6 years (range: 2 mo–68.8 years) for ILI cases, 30.7 year (range: 5–93.6 years) for SARI adult cases, and 1.0 year (range: 2 mo–4.96 years) for SARI child cases. The overall proportion of influenza positive was 10.8% (71/659) with 12.1% (36/297); 18.9% (21/111) and 5.6% (14/251) for ILI, SARI Adult and SARI child cases, respectively. The proportion of influenza positive cases was similar in 15–49 years (46.5%) and <15 years (44.9%) age groups (Table 1).

**Table 1:** Demographic and epidemiological characteristics of influenza cases in Rwanda from July 2008– May 2010.

Parameters	Influenza positive during inter-pandemic Period (n = 659) n (%)	Influenza positive during pandemic period (n = 2045) n (%)	p-value
**Age**			
Median (Range)	21.1y (3mo–93y)	19.4y (5mo–62y)	NS
<6months	4 (5.6)	19 (3.6)	<0.001
6–23 months	13 (18.3)	49 (9.2)	
24–59 months	3 (4.3)	54 (10.2)	
5–14 years	13 (18.3)	141 (26.5)	
15–49 years	33 (46.5)	257 (48.3)	
50–64 years	4 (5.6)	10 (1.8)	
≥65 years	1 (1.4)	2 (0.4)	
**Sex**			
Female	36 (50.7)	275 (51.7)	0.06
**Co-morbid conditions**			
Asthma	1 (1.4)	4 (0.8)	<0.001
Chronic breathing problems	3 (4.2)	167 (31.4)	
Chronic cough	0 (0.0)	3 (0.6)	
Cardiac disease	3 (4.2)	8 (1.5)	
Recurrent chest pain	3 (4.2)	1 (0.2)	
Chronic malaria	0 (.0)	3 (0.6)	
Diabetes	0 (0.0)	2 (0.4)	
Chronic malnutrition	0 (0.0)	4 (0.8)	
**Other**			
Pregnant	1 (1.4)	3 (0.6)	<0.001
Office worker	0 (0.0)	28 (5.3)	
Student	0 (0.0)	206 (38.7)	
Children at home	13 (18.3)	235 (44.2)	
Contact with a similar illness in last 3 weeks	2 (2.8)	116 (21.8)	

**Figure 3:**
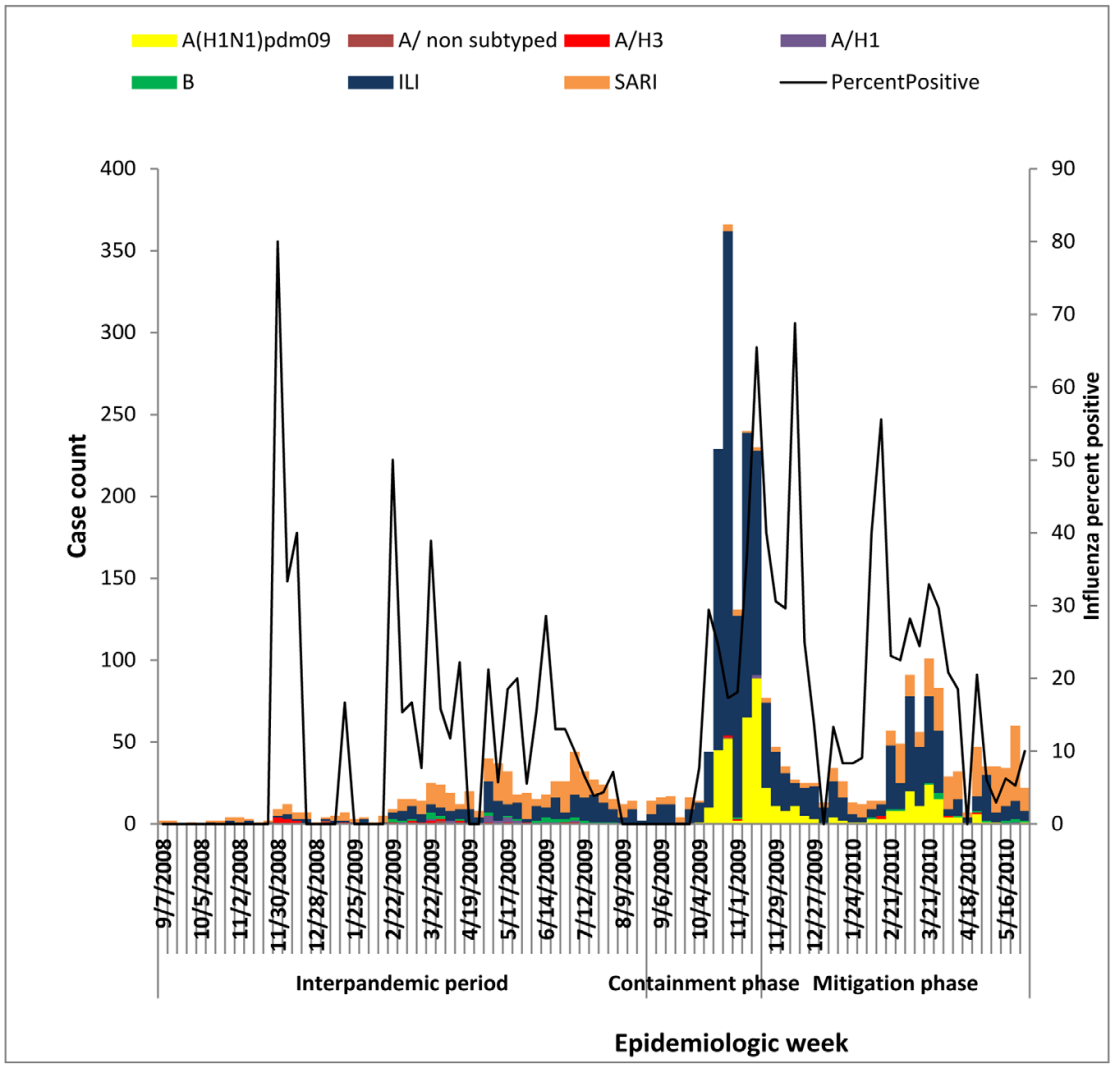
Case classification and influenza percent positivity by week in Rwanda. From July 2008– May 2010.

Of all influenza cases detected during the surveillance period, 56.3% (N = 40) were type A and 43.7% (N = 31) were influenza B viruses. The monthly trends of influenza activity revealed that the majority of influenza in 2008 season (July-December) was caused by Influenza A(H3) viruses. In 2009 season (January-December), initially, influenza A(H3) viruses predominated with some co-circulation of Influenza A(H1) and B viruses, but since October, A (H1N1) pdm09 largely predominated over other influenza viruses with limited co-circulation of influenza A(H3) and Influenza B (Figure 3).

### Pandemic Period

From October 9, 2009 to May31, 2010, 2,045 nasopharyngeal and oro-pharyngeal specimens were submitted to the NRL for testing. Of these, 69.7% (n = 1426) were ILI, 9.3% (n = 191) were SARI Adult and 21% (n = 428) were SARI Child cases. In total, 26% (n = 532) of specimens tested positive for influenza. Of these 96% (n = 510) were influenza A and 4% (n = 22) were influenza B. Of cases testing positive for influenza A, 96.8% (n = 494); 3% (n = 15) and 0.2% (n = 1) were A(H1N1)pdm09, Seasonal A(H3) and Seasonal A(Unsubtyped) respectively). The percentage of confirmed influenza cases due to A(H1N1)pdm09 was highest in the age group of <15 years (53.2%) compared with other groups (Tables 1 and 2).

**Table 2:** Distribution of influenza type and subtypes by age group during pandemic period in Rwanda from October 2009– May 2010.

Influenza	<6 mo	6–23 mo	24–59 mo	5–14 y	15–49 y	50–64 y	> = 65 y	Total
A(H1N1)pdm09	19	38	51	139	237	8	2	494
A/H3	0	2	1	0	10	2	0	15
A/Unsubtyped	0	0	0	1	0	0	0	1
B	0	9	2	1	10	0	0	22
Total	19	49	54	141	257	10	2	532
Percent	3.6	9.2	10.2	26.5	48.3	1.8	0.4	100

mo  =  months; y  =  year.

Among all influenza positive cases during the pandemic period, 26.9% (143/532) were among groups known to be at high risk of influenza-associated complications, including age <5 years (23%) and chronic breathing problems (31.4%) (Table 1).

Among all positive influenza cases, the most common symptoms at illness onset were cough (88.2%), fever (85.2%), headache (71.8%), lethargy (69.7%), sore throat (57.9%) followed by difficulty breathing (32.5%), muscle pain (32.3%), dizziness (30.1%), nausea (27.6%), vomiting (16.7%) and diarrhea 8.3% (Table 3). The median duration between onset of symptoms and health facility presentation of laboratory confirmed cases was three days (Range: 1–14 days) versus three days (range: 1–21 days) for influenza negative cases. In total, 85% of patients presented within seven days of illness onset.

**Table 3:** Clinical characteristics of positive and negative influenza cases during pandemic period in Rwanda from October 2009– May 2010.

Parameters	Influenza negative n (%)	Influenza positive n (%)	p-value
N (%)	1513 (74.0)	532 (26.0%)	
**Reported symptoms**			
Fever	1122 (74.2)	453 (85.2)	<0.001
Cough	1244 (82.2)	469 (88.2)	
Nausea	229 (15.1)	147 (27.6)	
Vomiting	297 (19.6)	89 (16.7)	
Headache	713 (47.1)	382 (71.8)	
Diarrhea	139 (9.2)	44 (8.3)	
Lethargy	925 (61.1)	371 (69.7)	
Difficulty breathing	678 (44.8)	173 (32.5)	
Muscle pain	245 (16.2)	172 (32.3)	
Dizziness	247 (16.3)	160 (30.1)	
Sore throat	680 (45.0)	308 (57.9)	
**Symptom duration relative to presentation in days**			
Median days (Range)	3 (1–21)	3 (1–14)	<0.001
0–3days	803 (53.1)	297 (55.8)	
4–7days	485 (32.1)	158 (29.7)	
>7days	44 (2.9)	11 (2.1)	
**Vital signs**			
Median °C Temperature (Range)	38.2°C(37–41.6)	38°C (36.9–40)	
Median admission pulse oximetry (Range)	97(85–100)	98(88–100)	
Median breathing frequency (Range)	32(15–72)	24(16–70)	

In total, 58 (12%) cases were hospitalized with mean duration of hospitalization of five days (Range: 2–15 days) and all cases recovered. Overall, 339 (68.6%) laboratory-confirmed cases received oseltamivir in all healthcare settings as curative and preventive treatment. On October 31, 2009 and November 6_,_ 2009, the second and third index cases were confirmed outside of Kigali. As of May 31, 2010, transmission of pH1N1 had been confirmed in 19 of 30 administrative districts in Rwanda and in all 5 provinces. Nine districts with the greatest number of cases accounted for over 92% of all confirmed cases. Six of these districts are home to sentinel sites and one is in Kigali City; the two other districts are more remote and in proximity to major border crossings with Uganda and the Democratic Republic of Congo. Presumptive transmission settings included: households (76.1%, 376/494), schools (41.7%, 206/494), and health facilities (10.1%, 50/494), prisons (5%, 24/494), solidarity camps (0.4%, 2/494) and foreign countries (1.2%, 6/494).

Attack rates of laboratory-confirmed cases during the containment phase ranged from 2.4% (42/1,750) to 3.8% (22/584) in schools and 1.6% (10/617) in the health facility attended by the index case. The proportion of cases originating from districts with sentinel sites was two-fold higher than those from non-sentinel site districts. The share from sentinel sites increased from 65% to 75% of all positive cases from containment to mitigation phase, while it decreased from 35 to 25% in non-sentinel site Districts (Table 4).

**Table 4:** Laboratory-confirmed A (H1N1) pdm09 by response phase and collection site in Rwanda from October 2009– May 2010.

Collection site	Containment	Mitigation	Total
	# (%)	# (%)	# (%)
Sentinel Site Districts*	178 (64.5)	163 (75)	341(69)
Non-Sentinel Site Districts±	98 (35.5)	55 (25)	153(31)
Total	276 (100)	218 (100)	494 (100)

* n = 6; Districts with an ISS site;

± n = 24; Districts without an ISS site.

## Discussion

The A(H1N1)pdm09 outbreak occurred in Rwanda at the beginning of the normal influenza season period and three months after the detection of the first cases in neighboring countries (Kenya, Tanzania, Uganda). Prior to the outbreak, any increased influenza activity in the surveillance system could have identified the outbreak. Instead, the index case was detected in a non-sentinel hospital, suggesting that sentinel surveillance may not be the best system to promptly detect the first case. The trigger of outbreak detection was the guidance provided by the Ministry of Health to the index case to seek care, and have nasopharyngeal and oropharyngeal swabs taken and tested for A(H1N1)pdm09 virus infection.

Following the initial pH1N1 detection, control measures such as airport screening of incoming passengers, promotion of hand hygiene, use of facial mask, and self-isolation of laboratory-confirmed cases were implemented at the beginning of the outbreak. However, these measures did not prevent the spread of pH1N1 in the general population. The containment phase investigation documented at least two additional likely introductions of pH1N1 to Rwanda; these laboratory-confirmed cases likely acquired infection from external settings. Moreover, the virus spread among children was related to school and social contact of the index case’s children with classmates and other students in Kigali. Having children at home and contact with a patient with a similar illness were the exposures more frequently identified among laboratory-confirmed pH1N1 cases compared to seasonal influenza patients (p<0.0001).

As mentioned earlier, during the pH1N1 outbreak the age group<15 years old had the highest proportion (53.2%) of influenza positive cases compared to the inter-pandemic period (44.6%). This is probably due to the demographic structure of Rwandan where more than 45.9% are <15 years [1], [2]. The pandemic strain has co-circulated with seasonal A(H3) and influenza B; but seasonal A(H1) has no longer circulated since the detection of A(H1N1)pdm09 (Figure 3).

During the shift from containment to mitigation phase, laboratory sampling methods were changed to selectively target most at risk and severely ill cases. Thus, a higher proportion of laboratory-confirmed influenza was expected during the mitigation phase. Instead, a slightly lower proportion −23.4% (218/931) - was reported compared with 24.7% (276/1114) –during the containment phase. The mitigation strategies implemented during the containment phase such as school closure and systematic distribution of oseltamivir to all suspect and laboratory confirmed cases may have helped to reduce viral transmission. It is also possible that the mitigation phase took place when the most susceptible population had already been exposed, and the rates in the general population had begun to decline.

Overall, disease severity was relatively mild with 70% of cases classified as ILI, 12% hospitalized, and no deaths. These findings are consistent with other reports from the region and internationally [8], [9], [13]–[15]. Our findings demonstrated that clinical symptoms and duration for pH1N1 virus infection were similar to what has been described for normal seasonal influenza illness [14]. We identified lower proportions of diarrhea (8.3% vs. 37%) and vomiting (16.7% vs.41%) compared to hospitalized pH1N1 patients in Kenya [15]. Symptoms significantly associated with A(H1N1)pdm09 positive cases versus negative cases were fever, cough, sore throat, nausea and muscle pain (p<0.0001).

Although Rwanda received 15,000 treatment courses of oseltamivir from WHO, this drug was used sparingly during the pandemic period to avoid drug resistance as the outbreak was considered to be a mild disease. Our attack rates were limited by the fact that they were only calculated within circumscribed communities (i.e.: schools, prisons) where it was possible to obtain denominator data. These attack rates are below the cumulative attack rate of 7.7% among all age groups in Lima, Peru [13], of 14.7% in Chicago among children aged 5–14 years [16] and of 43.4% in a community cohort study involving persons aged 5–14 years in Hong Kong [17].

Districts with influenza sentinel sites accounted for 69% of all positive confirmed cases and the share from the sentinel sites increased substantially from containment to mitigation phase (Table 3). These numbers demonstrated that the sentinel surveillance system served as the driving force behind the detection and sample collection of new cases of pH1N1. However, the lack of reporting from non-sentinel sites and districts also showed that the decentralized district hospital-driven strategy envisioned during the tabletop exercise was not fully realized. Although an initial attempt was made during the transition from containment to mitigation phases to train district hospitals on outbreak surveillance and management, the limited reporting reinforced the need to improve the functioning of influenza rapid response teams at the district level.

Our study has some limitations. We used different methods during containment (contact tracing and systematic testing) and mitigation phases (targeted testing) to identify cases and this may have introduced biases that reduce the representativeness of the findings with respect to the general population. The reported cases were limited to the hospitals where influenza surveillance is conducted and in hospitals where clinicians conducted systematic surveillance for pH1N1. Thus, additional cases of pH1N1 may have been missed at other health facilities and thus were not reflected in this report. Lastly, sentinel surveillance of influenza began too recently to provide robust data on seasonal influenza epidemiology and burden of disease to allow appropriate comparison with pandemic data (Table 1).

This study describes the epidemiology, clinical features, and the response to the outbreak of Influenza A(H1N1) pdm09 in Rwanda from October 2009 to May 2010. The outbreak occurred and peaked during the influenza season in Rwanda. Unlike seasonal influenza, children<15 years were the most affected. Our findings demonstrate that clinical symptoms and duration for A(H1N1pdm09 virus infection were similar to what has been described in the region and internationally. The outbreak helped to identify gaps in the incipient national influenza surveillance system. The lessons learned from the outbreak response also included the need to expand laboratory capacity to manage increased demand for specimen testing during epidemics; and to strengthen technical capacity at the district level to ensure the successful decentralization of outbreak management. Additionally, the quality of the integrated disease surveillance and response (IDSR) system must be improved in order to provide reliable surveillance data on current and future influenza outbreaks. Given the importance of ISS as a surveillance backbone during the pH1N1outbreak response, the system’s quality should be evaluated and improved to ensure timely detection of novel influenza strains with pandemic potential, and for better understanding of the epidemiology of seasonal influenza.

## References

[1] National Institute of Statistics of Rwanda (2002). Rwanda General Population and Housing Census.

[2] Rwanda Ministry of Health, National Institute of Statistics, ICF Macro (2009). Rwanda Interim Demographic and Health Survey 2007–2008..

[3] World Health Organization (2009). Influenza-like illness in the United States and Mexico.. http://www.who.int/csr/don/2009_04_24/en/index.html.

[4] World Health Organization (2009). New influenza A (H1N1) virus: global epidemiologic situation, June 2009.. Wkly Epidemiol Rec.

[5] World Health Organization (2009). Accessed 2009 Oct 14.. http://www.who.int/csr/disease/swineflu/en/index.html.

[6] Breiman RF, Nasidi A, Katz MA, Kariuki Njenga M, Vertefeuille J (2007). Preparedness for highly pathogenic avian influenza pandemic in Africa.. Emerg Infect Dis.

[7] CDC (2009). Introduction and Transmission of 2009 Pandemic Influenza A (H1N1) Virus –- Kenya, June–July 2009.. MMWR.

[8] World Health Organization (2009). CDC Real-time RTPCR (rRTPCR) Protocol for Detection and Characterization of Swine Influenza.

[9] Hayden FG, Belshe R, Villanueva C, Lanno R, Hughes C (2004). Management of influenza in households: a prospective, randomized comparison of oseltamivir treatment with or without postexposure prophylaxis.. J Infect Dis.

[10] Han K, Zhu X, He F, Liu L, Zhang L (2009). Lack of airborne transmission during outbreak of pandemic (H1N1) 2009 among tour group members, China, June 2009. Emerg Infect Dis, 15: 1578–1581.. http://www.cdc.gov/eid/content/15/10/1578.htm.

[11] Murray CJ, Lopez AD, Chin B, Feehan D, Hill KH (2006). Estimation of potential global pandemic influenza mortality on the basis of vital registry data from the 1918–20 pandemic: a quantitative analysis.. Lancet.

[12] Archer BN, Cohen C, Naidoo D, Thomas J, Makunga C (2009). Interim Report on Pandemic H1N1 Influenza Virus Infections in South Africa, April to October 2009: Epidemiology and Factors Associated with Fatal Cases.. Euro Surveill 2009.

[13] Tinoco Y, Razuri H, Ortiz EJ, Gomez J, Widdowson MA (2009). Preliminary population-based epidemiological and clinical data on 2009 pandemic H1N1 influenza A(pH1N1) from Lima, Peru.. Influenza and Other Respiratory Viruses, 3, 253–256.

[14] CDC (2008). Influenza: The Disease.. http://www.cdc.gov/flu/about/disease/index.htm.

[15] Mogoka Osoro E, Munywa P, Muthoka P, Gikundi S, Kariuki Njenga M (2009). Hospitalized patients with Pandemic (H1N1) 2009, Kenya. Emerging Infectious Diseases.. http://www.cdc.gov/eid.

[16] CDC (2009). 2009 Pandemic Influenza A (H1N1) Virus Infections -Chicago, Illinois, April–July 2009.. MMWR.58.

[17] Wu JT, Ma ESK, Cheuk Kwong Lee, Chu DKW, Po-Lai Ho (2010). The Infection Attack Rate and Severity of 2009 Pandemic H1N1 Influenza in Hong Kong. Clin Infect Dis. 51 (10): 1184–1191.. doi:10.1086/656740.

